# Stroke Mimicry: Unmasking a Brainstem Tuberculoma in a Young Patient

**DOI:** 10.1002/ccr3.70834

**Published:** 2025-08-29

**Authors:** Nithish Nanda Palanisamy, Dhiran Sivasubramanian, Virushnee Senthilkumar, Smrti Aravind, Sathwik Sanil, Karthick Balasubramanian

**Affiliations:** ^1^ Coimbatore Medical College Coimbatore India; ^2^ Department of Cardiology Children's Hospital of Philadelphia Philadelphia Pennsylvania USA; ^3^ Institute of Oncology, Sri Ramakrishna Hospital Coimbatore India; ^4^ Department of Critical Care Medicine Christian Medical College Vellore India

**Keywords:** antitubercular therapy (ATT), brainstem involvement, intracranial tuberculoma, stroke mimic, tuberculosis

## Abstract

Intracranial tuberculomas are rare brain lesions caused by the spread of *Mycobacterium tuberculosis* from distant sites, typically the lungs. They can mimic strokes, especially in young, immunocompetent individuals without typical tuberculosis symptoms. Early diagnosis and antitubercular therapy are crucial for recovery, particularly in regions where tuberculosis is endemic.

## Graphical abstract

A young male presented with stroke‐like symptoms ‐ progressive hemiparesis and cranial nerve palsies. MRI brain revealed a ring‐enhancing intrapontine lesion with perilesional edema, suggestive of tuberculoma. Early initiation of anti‐tubercular therapy led to complete recovery.

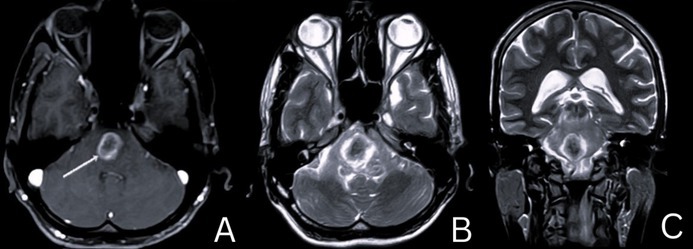

## 1 Case Presentation

**FIGURE 1:**
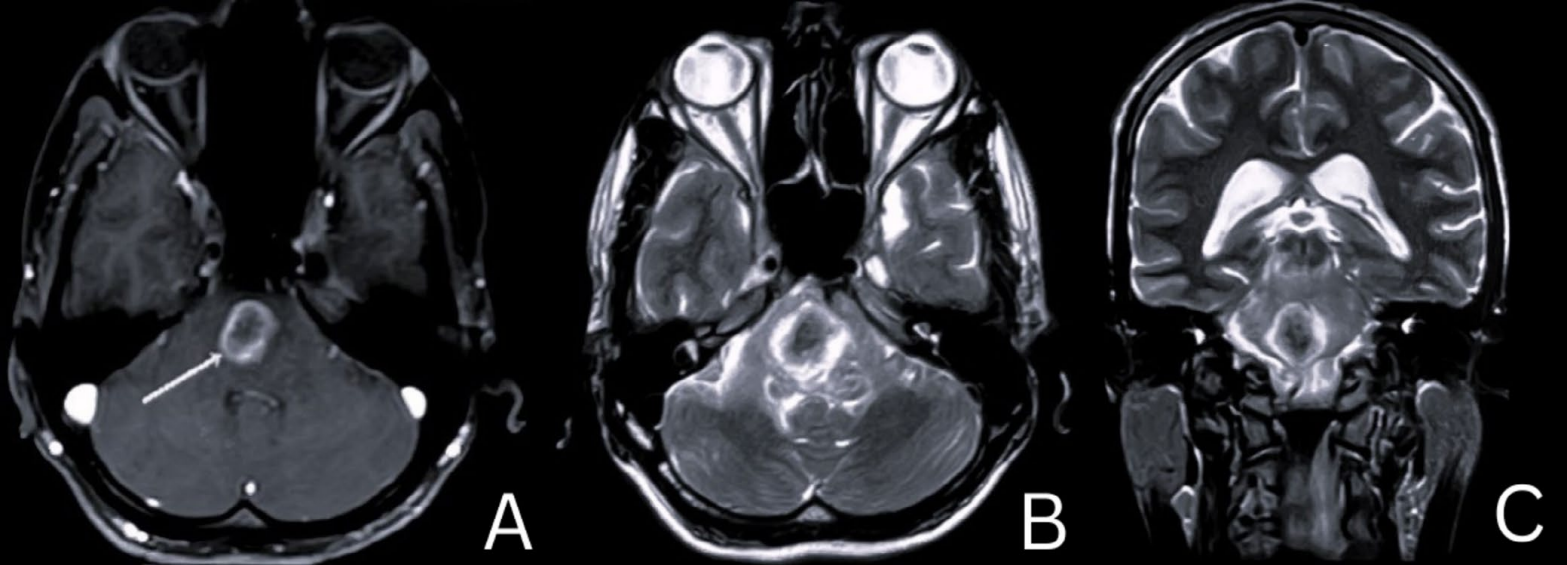
Gadolinium contrast‐enhanced magnetic resonance imaging (MRI) of the brain, (A) T1‐weighted axial image and T2‐weighted (B) axial, and (C) coronal view images showing a well‐defined ring‐enhancing lesion measuring 1.8 × 2.0 × 2.1 cm at the level of the pontomedullary junction with surrounding perilesional vasogenic edema.

**FIGURE 2:**
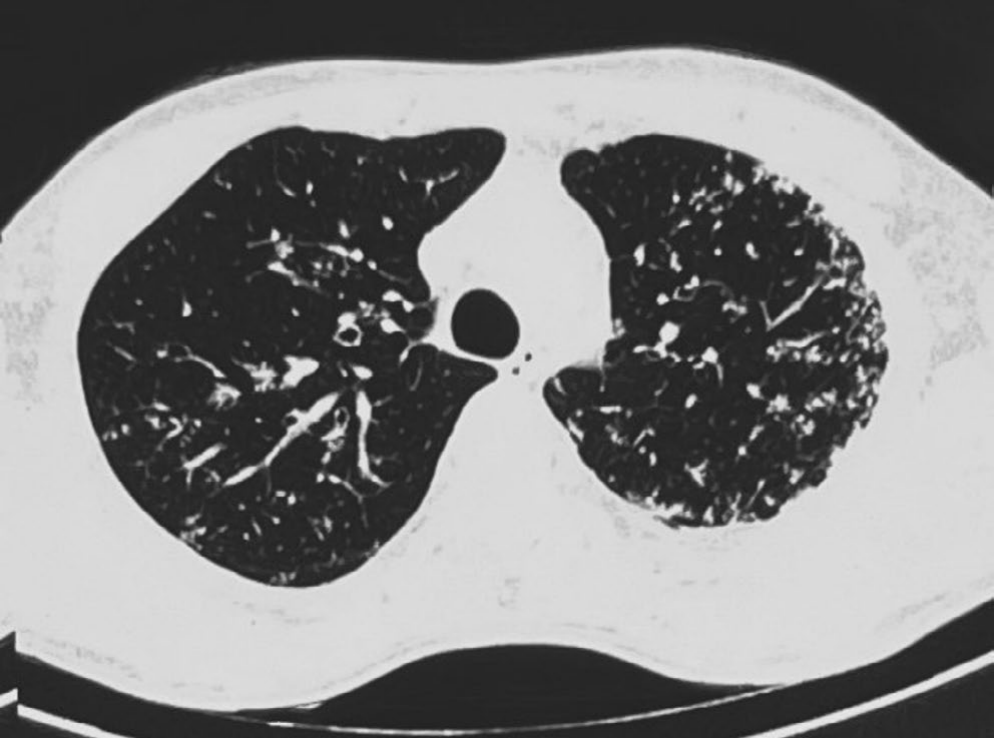
Computed tomography (CT) of the chest showing diffuse tree‐in‐bud opacities in bilateral lung fields associated with minimal pleural effusion.

**FIGURE 3:**
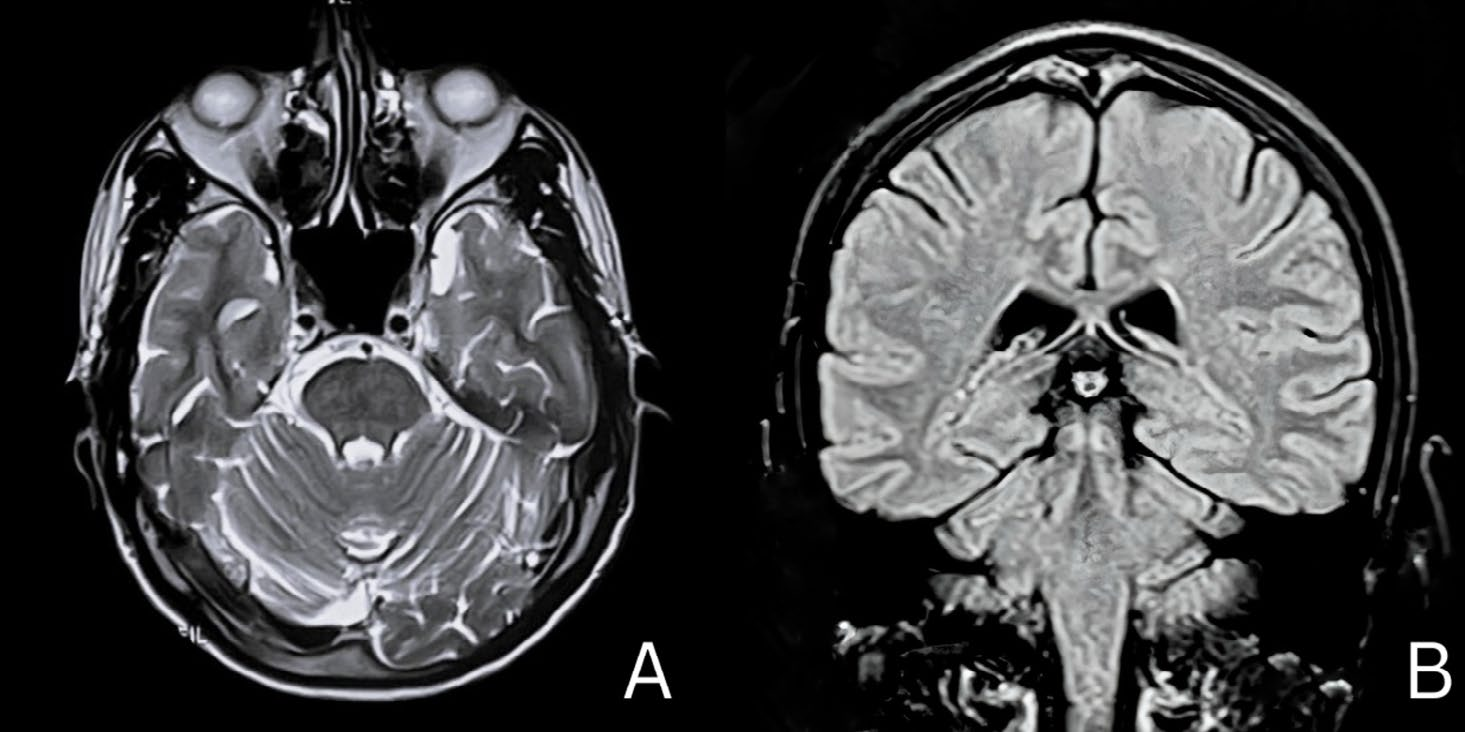
A follow‐up magnetic resonance imaging (MRI) of the brain, (A) T2‐weighted axial image and (B) T2‐weighted FLAIR coronal image showing no evidence of residual disease.

A 25‐year‐old Indian male presented to the emergency department with 3 weeks of progressive left‐sided limb weakness and difficulty maintaining balance. He also reported diplopia when gazing to the right and giddiness over the last 4 days. He denied systemic or respiratory symptoms and had no personal or family history of Tuberculosis (TB). He smoked and consumed alcohol but had no comorbidities. Upon examination, he was alert, oriented, and afebrile with no meningeal signs. Neurological examination demonstrated signs of brainstem involvement, including 6th cranial nerve palsy (lateral rectus weakness), 7th nerve palsy (facial muscle weakness), 8th nerve involvement (sensorineural hearing loss), and 9th and 10th nerve involvement (uvula deviation) on the right side. Corticospinal tract involvement was noted on the left side, with increased tone and exaggerated reflexes. Examination of the respiratory system showed occasional bilateral crepitations. Blood tests showed mild leukocytosis, elevated ESR/CRP, and HIV was negative. Magnetic resonance imaging (MRI) of the brain showed a well‐defined, lobulated intrapontine lesion with significant surrounding perilesional edema (Figure 1A). The lesion showed a hyperintense signal on postcontrast images, with a lipid‐lactate peak on MR spectroscopy, strongly suggestive of tuberculoma (Figure 1B,C). The lesion affected both the pons and medulla. A computed tomography (CT) of the chest revealed diffuse “tree‐in‐bud” opacities in both lung fields and a minimal left pleural effusion, suggesting pulmonary tuberculosis (Figure 2). Sputum AFB and CBNAAT came back negative. The patient was started on intravenous mannitol, dexamethasone, and antibiotics, along with a standard antitubercular therapy (ATT) regimen of Isoniazid, Rifampicin, Pyrazinamide, and Ethambutol with radiological confirmation of tuberculosis. On regular follow‐up, improvement was assessed clinically. Symptoms began to improve after 2 months of drug therapy. On completing 10 months of ATT, he presented with complete recovery. A follow‐up MRI revealed no evidence of residual disease (Figure 3).

## 2 Discussion

CNS tuberculomas are rare extrapulmonary TB lesions that can mimic ischemic stroke in young patients without traditional vascular risk factors. Brainstem involvement may present with focal neurological deficits resembling ischemic stroke [1]. The differential diagnosis for a young patient presenting with stroke‐like symptoms includes various stroke mimics such as tumors, toxic or metabolic disorders, infections, and demyelinating conditions [2]. However, characteristic tuberculoma findings of contrast‐enhanced MRI showing a ring‐enhancing lesion with perilesional edema and a lipid‐lactate peak on MR spectroscopy prompted further investigation, leading to the diagnosis [1, 3]. The differential diagnosis for ring‐enhancing lesions in the brainstem is broad; it includes infections (abscesses, neurocysticercosis), neoplasms (gliomas, metastases, primary CNS lymphomas) demyelinating diseases (tumefactive multiple sclerosis), and subacute infarcts. Treatment typically involves prolonged ATT and corticosteroids to reduce inflammation. Complications like hydrocephalus may require shunting or ventriculostomy. Intracranial tuberculomas are rare but should be considered in patients, especially in TB‐endemic regions. Early recognition and ATT can result in excellent outcomes even without systemic TB signs. This case reinforces the need to consider infectious etiologies in atypical stroke presentations.

## Author Contributions

**Nithish Nanda Palanisamy:** conceptualization, data curation, formal analysis, investigation, project administration, supervision, visualization, writing – original draft. **Dhiran Sivasubramanian:** conceptualization, data curation, formal analysis, funding acquisition, investigation, methodology, project administration, resources, software, supervision, validation, writing – original draft, writing – review and editing. **Virushnee Senthilkumar:** conceptualization, data curation, formal analysis, funding acquisition, investigation, methodology, project administration, validation, visualization. **Smrti Aravind:** conceptualization, data curation, formal analysis, funding acquisition, investigation, methodology, project administration, resources, software, supervision. **Sathwik Sanil:** conceptualization, data curation, formal analysis, methodology, project administration, supervision, validation, visualization. **Karthick Balasubramanian:** data curation, investigation, methodology, project administration, validation, visualization.

## Consent

Written consent for publication was obtained from the patient. The patient gave us consent for her medical information to be published in print and online, with the understanding that this information is publicly available.

## Conflicts of Interest

The authors declare no conflicts of interest.

## Data Availability

Deidentified patient data supporting this case report are included within the article and its Supporting Information; additional data (e.g., imaging or lab reports) are available from the corresponding author upon reasonable request, given ethical and confidentiality constraints.
